# Building on tradition: using the ADAPT-ITT model to develop a sexual health intervention from initiation rites of passage for rural young women in Southern Malawi

**DOI:** 10.3389/fpubh.2026.1761039

**Published:** 2026-04-20

**Authors:** Lucia Yvonne Collen, Gill Green, Eric Umar, Madalo Gloria Kalero Kuchawo, Sadandaula Rose Muheriwa-Matemba

**Affiliations:** 1 Department of Community Health Nursing, Kamuzu University of Health Sciences, Lilongwe, Malawi; 2 School of Health and Social Care, University of Essex, Colchester, United Kingdom; 3 Department of Health Systems and Policy, Kamuzu University of Health Sciences, Chichiri, Blantyre, Malawi; 4 Department of Human Development Nursing Science, College of Nursing, University of Illinois Chicago, Illinois, IL, United States

**Keywords:** adaptation, behavioral intervention, sexual and reproductive health, traditional initiation ceremony, young women

## Abstract

**Introduction:**

Young rural women are disproportionately affected by negative sexual and reproductive health (SRH) outcomes. However, few sexual health interventions for rural girls document the systematic process of leveraging the cultural tradition and integrating participant input in the development of SRH interventions. Aligning health interventions with familiar cultural practices increases both cultural congruency and relevance. This study aimed to adapt the Yao traditional initiation ceremonies for girls who have attained puberty (*Ndakula*) and those expecting their first child (*Litiwo*) into a sexual health intervention for girls who attain puberty in Balaka district in Malawi.

**Methods:**

This study employed an Ethnographic Participatory Action Research design, guided by the ADAPT-ITT [Assessment, Decision, Administration, Production, Topical Experts, Integration, Training, and Testing] model. This framework facilitated an iterative process of inquiry and intervention development. We conducted semi-structured in-depth interviews with purposively sampled women from three generations (young women aged 16–24 years, mothers, and grandmothers), traditional and religious SRH counselors, and participant observations of the traditional initiation rites ceremonies (*Ndakula and Litiwo*). We analyzed data using thematic content analysis. To ensure rigor and trustworthiness of data we employed methodological triangulation, peer debriefing, team analysis, and respondent validation. This comprehensive approach informed the co-creation and testing of a sexual health intervention tailored for rural girls who attain puberty. A descriptive evaluation of the intervention was done after one year of implementation.

**Results:**

Findings revealed important community assets including rich indigenous knowledge and a well-structured system for socializing girls and young women on sexuality and expected behaviors across different life stages, deeply rooted in the rituals and traditions of the Yao culture. Older women demonstrated remarkable depth of knowledge, creativity, and commitment in supporting the SRH of young women. At the same time, the study identified critical needs, including misinformation about SRH and women's reproductive physiology that promoted sexual activity during peak fertility periods. These identified assets and needs were collectively leveraged to co-create a culturally grounded SRH intervention for girls who reached menarche which the participants named: Partnership in Action: Health Improvement Intervention (PAHII). After one year of implementation, a descriptive evaluation of PAHII showed a 51.9% reduction in pregnancies and an 82.8% increase in the number of young women using family planning methods.

**Discussion:**

Findings support the importance of adapting culturally accepted traditional education, upholding healthy cultural practices, and leveraging deeply rooted values in research and interventions development and implementation. Further research is needed to examine the effectiveness of this sexual health intervention tailored for rural young women on a larger scale.

## 1 Introduction

Malawi has a predominantly young population with 64% of the total population under the age of 15, 18% under the age of 5, and only 3% above 65 years old (1, 2). Young rural women aged 10–24 face a disproportionate burden of early sexual debut, unwanted pregnancies, unsafe abortions, and childbirth complications (3–5), with the southern region being the most affected. Among sexually active adolescents in Malawi, about 14.5 % in the Southern region live with HIV infection, twice the prevalence observed in the Northern and Central regions of the country (6). Like many parts in sub-Saharan Africa, early sexual debut and childbearing are more common among the least educated, rural females and those from lower socio-economic households (7, 8). Mwale and Muula (9) in a study on sexual initiation among young people from different social backgrounds in Malawi also found that young people from the rural areas had earlier sexual debut, than those from the urban areas. A recent study in the southern Malawi among rural girls aged 16–24 also found that most girls become mothers by 17 years (10), with others getting married at the age of 13, and being mothers at age 14.

In Malawi, Balaka district, located in the southern region is one of the districts with higher rates of early sexual debut, early pregnancies, and childbearing. The recent Malawi demographic health survey (MDHS) 2024 showed that Balaka's adolescent pregnancy rate remains one of the highest in the country estimated at 41.8% (11) and early childbearing (women aged 15–19 years who had at least one live birth) was at 35.1% higher than the national level which is at 32% (11). In addition, about 32% percent of the women in Balaka reported not using any contraceptive method in the year preceding the survey (11). The higher rates of early childbearing in rural Balaka are partly attributed to the broader social acceptance of teenage pregnancy as it is perceived as a source of pride mostly for the mothers of teens because it reflects the maturity of their daughters and increases their social standing in the community (12–14). Research has also linked early sexual debut to riskier sexual behaviors, including having multiple partners, lower contraceptive and condom use, and increased HIV incidence (15). However, countries like Uganda, which have seen a significant decline in HIV cases, attribute part of their success to delaying sexual debut among adolescents (16). Community based interventions that address the social mechanisms contributing to early sexual debut, early childbearing, unsafe abortions and HIV/STI risk in young rural women are therefore urgently needed, especially in the absence of school-based comprehensive sexual education programs for preteens (9, 17, 18). Currently, few pregnancy, HIV/STI-prevention interventions target rural adolescents (19, 20). Interventions that target early adolescent girls may provide opportunities to encourage delayed sexual debut and establish consistent condom use and other healthy sexual behaviors that reduce the risk of teen pregnancy, STIs, and HIV infection (18). Further, few reports systematically describe the process of eliciting input from multiple stakeholders in the rural community to culturally tailor content for rural girls at this developmental stage. To promote program acceptance and support, and to strengthen future program sustainability, our study suggest that systematically engaging community stakeholders to inform program content and structure is crucial (21).

Local knowledge is vital for designing and implementing effective interventions. Evidence suggest that local knowledge offers practical, contextually relevant solutions, wisdom and insights that help ensure decisions are not only technically sound but also socially and culturally appropriate (18), making interventions become more responsive to the realities of the populations they aim to serve. Recognizing this value, the growing evidence places greater emphasis on integrating stakeholder perspectives throughout the development and implementation process (22, 23). However, over the years, rural women's input has not been adequately incorporated into interventions and programs which affect their health and wellbeing, contrary to the WHO Alma Ata declaration which states that, people have the right and duty to participate individually and collectively in the planning and implementation of their health care (24). Malawi adopted the 1978 Alma-Ata Declaration, to guide primary health care services (24). In line with this principle, rural women in Malawi were engaged in the process of adapting a traditional initiation ceremony into a sexual health intervention for young women.

### 1.1 Traditional initiation ceremonies in Malawi

Traditional initiation ceremonies differ across ethnic groups and between genders, serving as important cultural milestones that mark the transition from childhood to adulthood. These rituals impart moral values, social norms, and knowledge about future roles within the community (25). Among girls, the most common initiation rite is known as *Chinamwali*, a ceremony designed to transform girls into women by teaching them the responsibilities of adulthood and preparing them for marriage. During Chinamwali, girls undergo a period of seclusion under the guidance of older women known as *Anamkungwi* (Traditional Initiation Counselors). These counselors provide instruction on good manners, sexuality, hygiene, and respectful interaction with adults. The ceremonies often include traditional dances performed by women, symbolizing unity and celebration. Historically, some communities practiced a ritual of sexual cleansing involving a man known as *fisi* (hyena) (25), however, this practice has been outlawed in many parts of Malawi due to health and human rights concerns (26). Among the Yao people, who are one of the major ethnic groups in Malawi, a girl is expected to undergo three main initiation rites during her lifetime: *Mzondo, Ndakula, and Litiwo*. These ceremonies are conducted by special Sexual and Reproductive Health (SRH) instructors known locally as *Anakanga*, the Yao term for traditional initiation counselors. Each girl is accompanied by a mentor, someone close to her, who provides emotional and practical support throughout the process. Other women also participate by offering guidance, though men and uninitiated women are not permitted to attend, except during Litiwo, where all mothers are welcome (27). The timing and duration of these ceremonies vary according to the developmental stage of the girl. *Mzondo* is performed before menarche, typically when the girl is between 7 and 10 years old and lasts about three to four weeks. Ndakula takes place after menarche to prepare the girl for marriage, while Litiwo is conducted during pregnancy to prepare young women for childbirth, lasting from one to seven days. These ceremonies are often cherished by participants, as they represent a valued cultural tradition and a source of pride. Parents or guardians usually initiate the process and provide the necessary financial and material support required for the ceremonies.

This paper describes the process of adapting puberty and pregnancy initiation rites for young women in Malawi, focusing on how traditional practices can be aligned with modern health and education goals while preserving their cultural significance. This study provides a distinctive and innovative contribution by examining the construction of SRH knowledge and communication practices through a non-Western, intergenerational lens encompassing young women, mothers, and grandmothers in rural communities. Grounded in African feminism (28), the study foregrounds women's voices and lived experiences as central to understanding how SRH knowledge is produced, transmitted, and negotiated across generations within specific cultural contexts. By integrating Decoloniality theory (29), the paper advances a critical departure from dominant Western paradigms of SRH research (3–5, 30). It acknowledges the plurality of lives, lived experiences, cultures, and indigenous knowledge systems, positioning them as legitimate and essential sources of understanding rather than as peripheral or subordinate. This theoretical framing not only challenges epistemic hierarchies but also illuminates alternative ways of knowing and communicating about SRH issues that are rooted in local traditions and social relations. Together, these perspectives offer a novel framework for reimagining SRH discourse, one that values indigenous epistemologies, intergenerational dialogue, and feminist praxis as integral to advancing culturally grounded and contextually relevant health research.

## 2 The ADAPT-ITT Model

The ADAPT-ITT model (31) is a systematic, eight-step process for adapting evidence-based interventions (EBIs) to be more suitable for a different population, setting, or modified strategy. This framework involves assessing the target population, making decisions about which interventions to use, adapting the intervention through pre-tested methods, producing a new plan, involving topical experts, integrating information, training personnel, and finally, testing the adapted intervention for efficacy. The model's eight stages systematically integrate community insights to provide culturally relevant, scientifically sound, and effective interventions that address the needs of the target population (31, 32). This model has been applied to various HIV prevention efforts using quantitative and qualitative methods (31, 32), and within the Community Based Participatory Research (CBPR) (33). Using the ADAPT-ITT model to adapt a culturally accepted tradition, allows for the integration of cultural values and community perspectives into an intervention. This approach enhances cultural relevance and acceptability, which can improve community engagement, trust, and intervention uptake (34). Additionally, ADAPT-ITT incorporates iterative feedback and evidence-based strategies to refine the tradition in a way that aligns with public health goals while maintaining its cultural significance (31). Over time, this process can generate empirical support for the adapted intervention, bridging the gap between cultural practices and evidence-based approaches. The ADAPT-ITT model fosters active collaboration between the target population and investigators, ensuring the development of a culturally responsive and inclusive study (32). A key advantage of this approach is the early engagement of the community, which helps build trust particularly among populations with a history of medical and research mistrust while also enhancing the likelihood of sustained participation in the intervention (32, 35).

## 3 Methodology

### 3.1 Design

**Figure 1:**
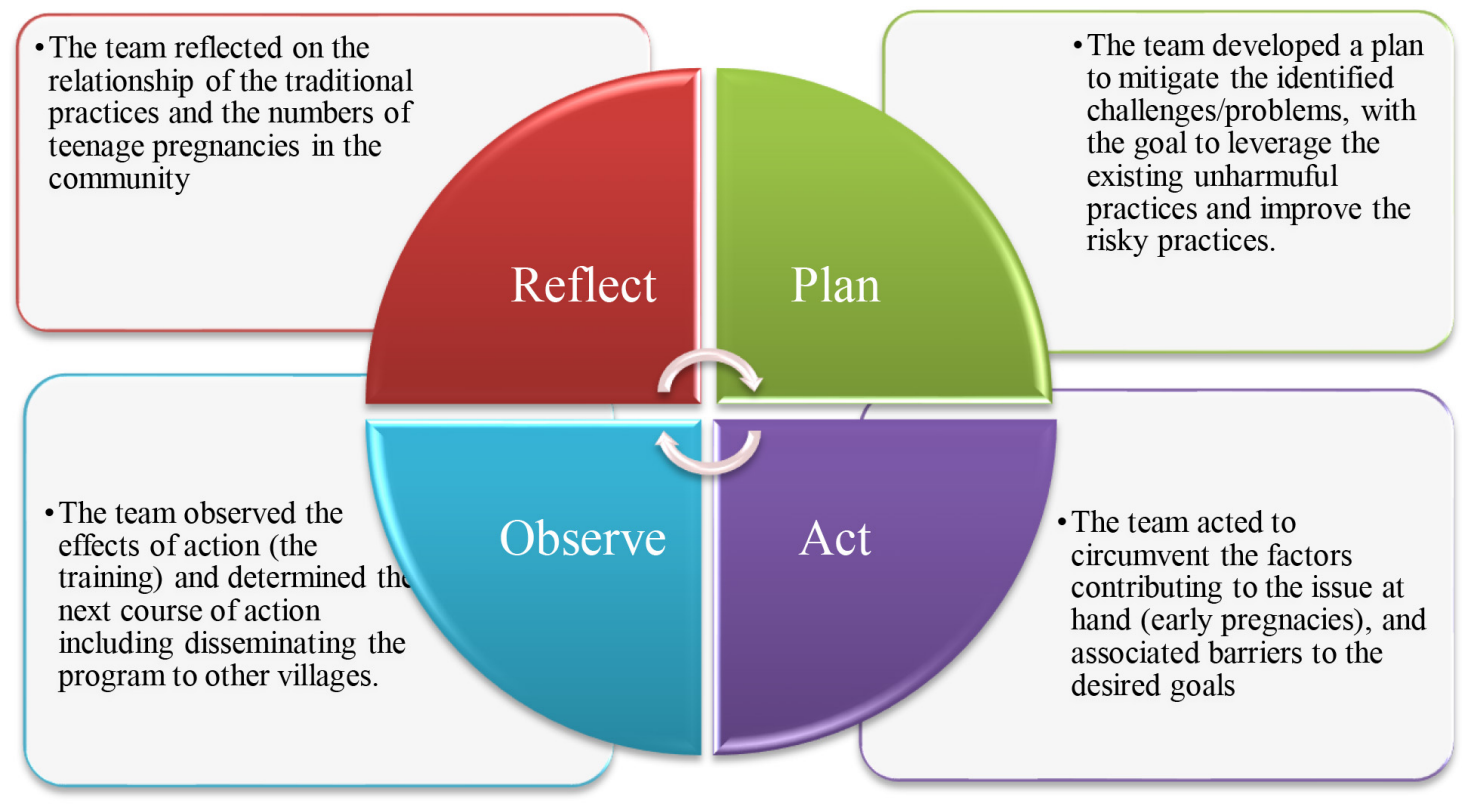
A diagram illustrating the four action steps of Participatory Action Research adapted from Kemmis et al. (36), Willis and Edwards (37) as applied to the adaptation of a traditional initiation ceremony into a sexual and reproductive health intervention for young women in rural Malawi.

This study which focuses on the adaptation of the traditional initiation ceremony into a SRH improvement intervention for young women, was part of a larger study that used Ethnographic and Participatory Action Research design whose purpose was to explore the views of rural women (young women, mothers, and grandmothers) and influencing factors on SRH communication practices, contexts that shape the intergenerational SRH communication experiences, and assist the rural women to act in solving SRH problems and issues among themselves by promoting indigenous knowledge. This part of the study utilized the ADAPT-ITT model (31) which incorporated all eight phases: (1) Assessment, (2) Decision-making, (3) Administration, (4) Production, (5) Topic experts, (6) Integration, (7) Training, and (8) Testing of the adapted intervention. Under each phase the investigators followed the four principles of reflect, plan, act and observe (36). This study was conducted in collaboration with the community members. Various stakeholders including the Health Surveillance Assistants (HSAs), teachers, community village chiefs, caregivers and other influential religious and community leaders played a key role in informing the study direction, and their input was used to reshape the research design, including structuring of the health improvement intervention. Connections with the community members fortified our commitment to prioritizing stakeholder engagement throughout adaptations. We used the ADAPT-ITT within the Ethnographic and Participatory Action Research to ensure a rigorous process for adapting the traditional sexual health education into a sexual health improvement intervention (33). Each phase of the ADAPT-ITT was guided by the four principles of the reflective and action learning, thus reflect, plan, act and observe (36, 37) as shown in Figure 1. After one year of implementing the intervention, we conducted a descriptive evaluation to determine if the new curriculum was put into use. The evaluation included reviewing the family planning register for the catchment population (Mbatamila Village Health Register), conducting informal interviews with new initiates, and observing an initiation ceremony to assess whether new knowledge had been integrated and harmful practices removed from the curriculum.

### 3.2 Ethical considerations

Approval to carry out the project was obtained from two institutional review boards in Malawi and United Kingdom (anonymized). Consent was obtained from all adult participants. Approval to conduct the study was also sought from the District Commissioner, District Health Office and the village chiefs who are the gatekeepers. In addition to assigning the participants numbers, during the interviews we used pseudonyms, which participants identified for themselves. To acknowledge participants' time and contributions, each was given a *chitenje*, a large rectangular piece of cloth commonly used by women in Malawi and many parts of Africa for various purposes, such as wrapping around the waist like skirt, draping over the shoulders, carrying babies, or tailoring into clothing. This token of appreciation was provided at the conclusion of the ethnographic participatory action research when the researcher was leaving the village. Participants were not informed about the compensation beforehand to minimize response bias and ensure that their participation was motivated by genuine interest rather than expectation of reward. Additionally, in Malawian culture, gifts given as a surprise are considered more meaningful and respectful, aligning with local customs of appreciation.

### 3.3 Sample

Using purposive and snowball sampling, young women, their mothers, and grandmothers were recruited to participate in individual in depth interviews and focus group discussion (FDGs). Eligibility for FDGs was being an active SRH traditional or religious initiation counselor. To participate in the study, young women had to be between 16 and 24 years old, as most adolescents become sexually active by age 16. This age range was selected because it represents a critical developmental period during which many girls may have transitioned through puberty, participated in *Ndakula, Litiwo* and other initiation ceremonies, and begun navigating sexual relationships. These experiences positioned them to reflect meaningfully on their SRH knowledge, practices, and communication patterns. Although eligibility began at age 16, the youngest participant enrolled was 18, because individuals under 18 either declined or did not meet eligibility criteria. Thus, participants had sufficient maturity and lived experience to engage in in-depth discussions about SRH, and the age gap that could reflect different sexual health needs was reduced. Mothers were included if they had a daughter aged 16–24, and grandmothers were eligible if they had a daughter who was the mother of a 16–24-year-old young woman, representing the custodians of cultural traditions and intergenerational knowledge transmission. Including these three generations allowed for the exploration of intergenerational differences and continuities in SRH communication and knowledge systems. Comparing perspectives across young women, mothers, and grandmothers provided insight into how cultural norms, gender expectations, and social change influence the evolution of SRH discourse over time. This design illuminated both the persistence of traditional beliefs and the emergence of new understandings shaped by education, modernization, and shifting gender dynamics. The traditional and religious counselors were from two villages, one predominantly Muslim and the other predominantly Christian. In the Christian village, both traditional and religious counselors participated in the study, while in the Muslim village, participants were predominantly traditional counselors, as Muslims do not have religious counselors. All participants were required to have prior experience with traditional initiation ceremonies and to complete an informed consent. The initial target sample size was 25, selected to obtain rich, nuanced, and comprehensive data from three generations of women. This target aligns with ethnographic research standards, which consider a sample of 6–10 participants adequate for generating in-depth insights, while emphasizing the importance of reaching data saturation (38). The final sample included 27 participants, as data collection continued until saturation was achieved, that is, when no new information or themes emerged from participants' narratives (39).

### 3.4 Recruitment

Both passive and active recruitment strategies were employed to identify and engage participants. Passive recruitment involved sharing information about the study through community networks, word of mouth, and collaboration with local organizations working with young women, allowing interested individuals to approach the study team voluntarily. Active recruitment was facilitated through personal connections and relationships within the community, supported by community research partners and HSA who directly informed potential participants about the study. As an ethnographic study, the first author resided in the village throughout the research period, which enabled continuous engagement and participant invitations through informal community interactions. Interested individuals were encouraged to contact the study team directly. Study staff then administered a screening form to assess eligibility, with all screenings conducted in person. Eligible participants underwent a detailed informed consent process, during which the study's purpose, procedures, and ethical considerations were explained. Those who agreed to participate provided written informed consent, while individuals unable to write indicated consent using a thumbprint. Each consenting participant also provided sociodemographic and contact information for study documentation.

### 3.5 Data collection

Data collection took place in real world settings, in the village where the participants resided. Interviews used open-ended questions, and were carried out in the environment that was comfortable and familiar to participants (40). We triangulated three data collection methods: in-depth interviews, focus group discussions (FGDs) and participant observations to uncover and explore diverse SRH knowledges, rituals, and traditions, with field notes serving as an integrative analytic tool, allowing for the synthesis of insights across data sources. We conducted a total of 20 in-depth interviews, two FGDs and two observations of traditional initiation ceremonies. Using the three methods was useful as it strengthened the credibility and richness of the findings by revealing both consistencies and variations between what participants said and what they did. This convergence and divergence of data added depth to the interpretation of participants' perspectives and behaviors (41). Combining observations and conversations is a powerful data collection strategy as it provides multiple views of each incident for a researcher to think about (39, 42). FGDs and in-depth interviews provided a good social context for gaining a deeper understanding and putting meaning to young women's sexual health experiences and perspectives (41). They also provided an opportunity for issues to be explored in detail, as the interviewer has the chance and time to ask questions and seek clarification of issues raised (41). Each interview lasted between 45–90 min and were audio recorded. The FDGs lasted for over 120 min. All interviews were audio-recorded. Data collection was done from April to November 2021.

### 3.6 Data analysis

We used Braun and Clarke (43)'s six-step thematic analysis framework to analyze and while integrating the principles of participatory action research (36, 37). The first author transcribed all audio-recorded data. To ensure accuracy of the transcripts, three of the transcripts, two for young women, one for traditional counselors were randomly selected and reviewed by qualitative research experts who cross-checked them for accuracy (39). Following familiarization with the data, a thematic framework which was informed by each set of transcripts, by each category of participant (young women, mothers, grandmothers, and counselors), was developed. This was guided by the research questions, objectives of the study and the major themes and concepts that emerged from each set of transcripts (43). This approach was fit for use in Ethnographic Participatory Action research, as it does not only organize and describe a data set but goes further to include interpretation of various aspects of the research topic. When all data had been coded, different codes were sorted into potential themes and organized all the relevant coded data extracts within the identified themes. A table was used to demonstrate the relationship of emerging themes and between different levels of themes (43). The sorting of the identified themes involved listing all the themes identified and re-grouping them into main themes and sub-themes or sub-categories (44). During data analysis, we employed four types of triangulation: methodological, data source, investigator, and theoretical (45). Methodological triangulation involved systematically comparing and integrating findings from in-depth interviews, focus group discussions, and participant observations to identify areas of convergence, complementarity, and contradiction. For instance, we examined the alignment between what participants reported in interviews and what was observed in the field to assess consistency between expressed and enacted behaviors. Data source triangulation involved analyzing data from three different participant groups (young women, mothers, and grandmothers) to explore whether similar themes emerged across groups. This process illuminated shared patterns as well as contextual differences, enriching the overall interpretation of findings. Investigator triangulation was achieved by engaging multiple researchers (LYC, GG, SRM, LN) in coding and interpreting the data. Comparing analytic memos and coding decisions helped minimize individual bias and enhanced the reliability and credibility of interpretations. Theoretical triangulation involved interpreting findings through multiple theoretical lenses, including ethnographic, participatory action, and cultural competence frameworks. Applying these perspectives uncovered different dimensions of meaning, deepened understanding, and highlighted alternative explanations within the data. This process was facilitated using Computer-aided qualitative data analysis software (CAQDAS) QSR NVivo. Themes were then reviewed, refined, and named. Memoing was done throughout the process which helped in defining the codes and themes. To begin addressing the identified gaps in sexual education, HIV/STI and pregnancy prevention for at-risk youth in Balaka, we developed a culturally tailored evidence-based HIV/STI and pregnancy prevention program.

## 4 Results

Table 1 shows the sociodemographic characteristics of the participants who took part in the in-depth interviews and in FGDs. All participants were females aged 18–80 and most of them were subsistence farmers. Of these participant, seven (six traditional initiation counselors and one religious initiation counselor) participated in FGDs, and their age ranged from 30–75 years. Our findings align with the eight phases of the ADAPT-ITT Model. Each phase responded to a key question that guided the adaptation process. Each phase of the ADAPT-ITT was guided by the four principles of the reflective and action learning, thus reflect, plan, act and observe (36, 37) as shown in Figure 1. Table 2 outlines the adaption phases, questions, methodology followed for each phase, results, and adaptation decisions.

**Table 1:** Socio-demographic characteristics of the participants.

Sociodemographic characteristic	Adults (*N* = 17)			Young women (*N* = 10)		
	**Range/M**	***n***	**%**	**Range/M**	***n***	**%**
Age	30–80/63.7			18–24/21.4		
Education						
Adult literacy (acquired reading and writing skills in adulthood)		1	5.9		–	–
Did not go to school		11	64.7		–	–
Junior primary school (grade 1–4)		2	11.8		2	20
Senior primary school (grade 5–8)		3	17.6		6	60
Junior high school (Form 1–2)		–	–		1	10
Senior high school (Form 3–4)		–	–		1	10
Marital status						
Single		–	–		1	10
Married		8	47.1		5	50
Divorced		3	17.6		4	40
Widowed		6	35.3		–	–
Religion						
Moslem		11	64.7		9	90
Catholic		3	17.6		1	10
Pentecostal church		3	17.6		–	–
Source of income						
Subsistence farming		13	76.5		1	10
Subsistence farming & herbalist		2	11.8		–	–
Casual work & farming		2	11.8		4	40
Business/farming		–	–		3	30
Housewife		–	–		2	20

**Table 2:** Application of ADAPT-ITT model to a sexual health intervention for rural young women in Balaka.

Phase and target question	Methodology	Results/Observations, and Decisions
1. **Assessment** a. What is the context of SRH among rural young women in Balaka? b. Who is involved in teaching sexual health education? c. What is taught and how? d. What is the role of traditional rituals and beliefs on SRH issues among the rural women like the Yaos in Malawi? e. What assets are available in the community that can enhance the implementation of the possible solutions f. What are the gaps or needs of the Mbatamila community?	1. Conducted literature review 2. Collected qualitative data 3. Conducted participant observation of two main traditional ceremonies for girls after attaining menarche (*ndakula*) and for the girls with their first pregnancy (*litiwo*).	Literature review •Yao people and the Islamic religion embrace traditions •Women elders are the teachers of SRH •There is a gap in knowledge on the influence of traditional rituals on SRH issues among the rural women like the Yaos in Malawi **Main problem of concern/Gaps/Needs** •Increased early pregnancies particularly following initiation ceremonies •Emphasis on using waist strings to prevent pregnancies •Incorrect information on the female reproductive health system, particularly menstrual cycle **Possible contributing factors** •Incorrect sexual health information mainly with regards to the reproductive cycle •Socializing young women to be very submissive in sexual health matters
2. **Decision** a. What evidence-based intervention and traditional practices and messaging needs to be adapted*?* b. What are the community generated solutions to the SRH needs?	Conducted a community meeting with the key personnel in the village to present findings, identify areas that need actions and possible solutions	•Decided to leverage a well-coordinated traditional practice of holding initiation ceremonies regularly to socialize young women and girls on sexual matters and other expected behaviors, at different stages of their life, each with its own dictates at family, community, and societal levels. •Decided to add the reproductive health information including menstrual cycle to the traditional initiation curriculum •Decided to adapt the Malawi Government Reproductive Health and Family Planning program
3.**Adaptation** How should the intervention be adapted?	Administered theater testing (pretesting) of the original initiation ceremony curriculum Rated the process and content Analyzed results of the theater test	•The traditional SRH traditional counselors conducted a mock initiation ceremony on young women who played a role of girls who just attained menarche and were undergoing initiation ceremony. •In collaboration with observers identified areas that needed adaptation
4. **Production** How do you produce adapted traditional initiation curriculum?	Compiled the adaptation recommendations	•Produced draft 1 of the adapted traditional initiation ceremony curriculum •Balanced priorities while maintaining fidelity to the core elements and underlying theoretic framework of the original EBI •Developed an adaptation plan •Develop quality assurance and process measures
5.**Topical experts** Who can help adapt the intervention, and what additional content should be included?	Identified experts knowledgeable in women's and adolescent sexual and reproductive health including family planning, teenage pregnancy, and STI/HIV prevention, and knowledgeable in Yao culture, CBPR, Implementation Science Research, and sociocultural and behavioral dimensions of humans and health	•Identified and actively involved experts in sexual and reproductive health, Yao culture and community health. •A designer was also consulted to assist with the development of the training manual and design
6. **Integration** What is going to be included in the adapted EBI that is to be piloted?	Integrated feedback from participants and topical experts Completed draft 2 of the PAHII manual Integrated the in-depth interview questions and measures that can help assess the new intervention.	•Integrated feedback from the experts and the participants in the adapted SRH curriculum and the training manual •Completed draft 2 •Reviewed interview guide and incorporated them in the adapted intervention and implementation protocol.
7. **Training** Who needs to be trained?	Trained traditional SRH counselors, grandmothers, mothers and young women and community health workers to implement PAHII Produced draft 3 of PAHII based on feedback	•Trained the facilitators of the traditional initiation ceremonies to implement the intervention. •Produced draft 3 based on the feedback from participants.
8. **Testing** Was the adaptation successful, and how did it enhance short term outcomes	Implementation of the intervention continued for a year, supervised by the community health worker, the HSA.	•Program was evaluated after one years •Number of women attending family planning increased by 82.8% while number of pregnancies decreased by 51.9%

### 4.1 Phase 1: assessment

The assessment phase was comprised of a literature review and qualitative data collection and analysis to assess the status and context of SRH among rural young women in Balaka district, including assets and needs. Results of the literature review showed that unlike some other tribes, the majority of the Yao people converted to Islam, a religion credited for not interfering with the traditional beliefs and customs of the people (46). Traditionally, the women elders are given the responsibility for teaching young people about cultural assumptions, expectations, roles and practices regarding sexual behavior (47). Nevertheless, the literature review showed a clear gap in knowledge on the influence of traditional rituals on SRH issues among the rural women including the Yao women in Malawi. Previous studies among the Yao people used qualitative approaches and were mostly conducted by a male researcher not indigenous to this tribe and often written from a Western perspective (3–5). Thereby warranting an alternative approach of ethnographic participatory action research and perspective, and specifically targeting three generations, as well as considering the gender and positionality of the researcher on the study topic. Analysis of the individual in-depth interviews, FGDs, and observations of the puberty and pregnancy initiation ceremonies revealed five major themes that informed the adaptation of the traditional initiation ceremony to form a sexual health intervention: (1) SRH assets, (2) sexual socialization of a young woman, (3) gender and power dynamics: sexual power imbalance, (4) personal and societal identity, and (5) intergenerational use of sexual health knowledge systems.

#### 4.1.1 SRH assets

The ethnographic experience, observations of puberty (*Ndakula*) and pregnancy (*Litiwo*) initiation ceremonies, and in-depth interviews revealed several assets within the Yao community that can be leveraged to strengthen SRH education for young women. These assets included the presence of traditional SRH counselors and a well-organized system of socializing girls and young women on sexual matters and expected behaviors at different stages of life, with each guided by specific norms at the family, community, and societal levels. Grandmothers and traditional counselors hold a revered position within the Yao community and are among the most trusted individuals. All the three generations (young women, mothers, and grandmothers) talked about the key role of grandmothers and traditional counselors in sex education. Zione a 24-year-old young woman said:

*I was advised by my grandmother before I attained puberty, when my breasts started growing my grandmother called me and started advising me that we do this [meaning pulling of the labia minoras which are believed to help with sexual pleasure] so that you can get married, without this your marriage wouldn't last long. “It's not proper for me to discuss with my mother about sexual health!”*

One mother also said:

*When I started menstruating, I told the elders [grandmother] and they gave me pieces of old cloth [traditional sanitary pads] to use and told me that after using, I should wash them and dry them and keep them somewhere where they could not be seen by anyone. In addition to hygiene, I was told that I should not have boyfriends. I should wait until I was a fully grown up and I met someone who wanted to marry me* (Sumini a 48-year-old mother).

One grandmother added:

*I stained myself. Then my parents took me to a grandmother who started advising me, telling me, it was normal process, a sign that I am grown up* (Edina, a 70-year-old Grandmother).

Most participants reported being less embarrassed in the presence of their grandmothers than their mothers whose advice mostly came as a warning about pregnancy and STIs including HIV/AIDS, as evident in this quote:

“*You are not ashamed when talking to your grandmother. My mother! aaah no, but my grandmother.” You are never free with your mother, but with your grandmother and you can tell your grandmother anything without feeling ashamed* (Awetu a 20-year-old young woman).

Unlike grandmothers, findings showed that traditional counselors used initiation ceremonies to teach sexual education. Findings revealed the depth of knowledge and creativity that older women and traditional SRH counselors bring to supporting young women, particularly in areas such as menstrual hygiene and sexuality. These practices reflect the complex and nuanced nature of indigenous knowledge systems, which are often embedded in rituals and cultural traditions.

*Anankungwi [traditional counselor] are the most respected people in our community. Every parent wants their children to be advised by them. When I attained menarche, after finishing menstruating, my mom invited the Anankungwi then we went to the river to give me pieces of advice. They drew three* lines red, black, and white drawn on my thighs. *After that they asked me: “what did you see?” I said, “I saw this,” [pointing at the red line that symbolized menstrual blood] when I finished, I saw this” [pointing at white line that symbolized discharge after menstruation*] (a 70-year-old Grandmother: Edina).

This was acknowledgment of the transition of a girl child from childhood to adulthood, which is seen as a crucial period in a woman's life, during which sexuality, gender norms and expectations are learned and regulated by the society.

Participants also highlighted the discipline and respect instilled by initiation counselors, as well as their knowledge of SRH, as key reasons for one to undergo initiation ceremony.

*It [Ndakula (puberty initiation ceremony] has helped me on respect and behavior. My family life is going on well because of the advice I received. So is good to attend Ndakula. My marriage is going on well* (Linesi, a 21-year-old young woman).

Hadija a 46-year-old mother also added:

*The advice I received during Ndakula was used in my family. I was doing what I was told for my husband and my family was good. I have followed the advice until today. I feel the advice I was given during Ndakula has helped me in my family life*.

The traditional counselors' authority also stemmed from the belief that they possess the power to protect initiates from evil spirits and people with ill intentions. The high regard for the sacred ritual performed to protect the girls from individuals with harmful or ill intentions was revealed in the findings from the observation of the puberty initiation ceremony. This ritual is deeply respected within the community and is regarded as an essential act that ensures the girls' safety and spiritual wellbeing throughout the initiation process and is one of the motivation factors for parents to send their children for initiation ceremony. The reverence shown during this ritual underscores its cultural significance. Community members view it not merely as a symbolic act but as a vital spiritual safeguard that connects the girls to ancestral protection and affirms the authority and wisdom of the traditional counselors who perform it. The high regard for this ritual was also reflected in the narrative of one traditional counselor:

*The Ndakula ceremony starts at a place where the household waste is burnt, with one of the counselors bringing two pieces of a broken calabash and adding a drop of water on each plus something that looked like ash. The paste is then applied to the girls' breasts, arms, wrists, ankles and feet. Then the empty pieces of the calabash were put on the ground and girls are asked to break them with the right foot. This often triggers jubilation and ululation because this ritual is meant to protect girls from any harm, by making them invisible to people with ill thoughts as well as witches and wizard. So parents and chiefs want their girls to be protected, that is why every girl has to undergo Ndakula* (Abiti a 30-year-old traditional counselor trainee).

The study also identified other sources of SRH information available through government initiatives, including schools, health facilities, and the presence of local community health workers HSAs. Despite these resources, their impact on preventing negative SRH outcomes appeared limited, as young women primarily accessed health facilities for antenatal, delivery, and postnatal care, often after becoming pregnant, and not for prevention of early pregnancies, HIV infections and other STIs. Women in this community also had not abandoned their indigenous ways of thinking, knowing, and living. These traditional philosophies continued to guide initiation ceremonies and influence SRH decision-making and behaviors.

#### 4.1.2 Sexual and family socialization of a young woman

Findings revealed that traditional initiation ceremonies are an important source of SRH information among young women. Emphasis was put on care and hygiene during menstruation and sexuality.

*During Ndakula, the girl is advised on how to live with her husband, for instance when she is menstruating, she is told on how to take care of herself and her husband. In terms of family life, she is also advised on what to do once she finds a marriage suitor. She is also counseled on the dangers of unplanned pregnancy and if the girl is smart, she keeps all this in her head (Atuweni a 41-year-old mother:)*.

Additionally, teaching girls sexual techniques by traditional counselors was an important component of the puberty initiation ceremony. However, girls were taught sexual techniques while simultaneously instructing them to abstain from sex until a marriage suitor was found. This message created confusion to young women as well as mothers. One mother said:

*They are told not to indulge in promiscuous behaviors, but to help them when they find a marriage suitor, you must do this and that [referring to the sex demonstrations]. But today's girls, when you tell them this, it's like you are giving them a ticket to promiscuous behaviors. But the advice is meant to help them with their marriage, so that they do not experience any problem* (Esmie a 50-year-old mother).

This dominant message lacked clarity, as the girls were not taught what qualifies someone as a suitor or how to recognize one. This contradiction has the potential to confuse young women or encourage them to experiment sexually in order to perfect their skills in preparation for marriage, increasing their risk of early pregnancy, child marriage, and sexually transmitted infections, including HIV/AIDS. Furthermore, advice suggesting that sex is safe at any time outside menstruation, without reference to ovulation or fertility awareness, was also found to be misleading.

The narratives also revealed significant shortcomings in the sexual socialization of young women. For example, some traditional SRH counselors provided biologically inaccurate guidance, such as instructing young women to wait for the appearance of a white, watery, and slippery vaginal discharge after menstruation before engaging in sexual activity, claiming it indicated a safe time for intercourse. This type of misinformation can unintentionally increase the risk of teenage pregnancies. Aida, an 18-year-old young woman said:

*Then the Anankungwi told me; “when you finish menstruating and you see this [whitish creamy stuff], don't sleep with a man, the man dies, or he develops hydrocele. You sleep with a man when you see clear watery and slippery discharge like this [pointing at another watery slippery substance they had prepared* (a type of discharge that signify ovulating).

#### 4.1.3 Gender and power dynamics: sexual power imbalance

Power imbalance in sexual relations between women and men was also identified. Girls were taught to please men at all costs. What was common among all the generations (young women, mothers, and grandmothers) was the instruction to never say no to sex when their husbands wanted to engage in sexual activity except when menstruating or unwell, because by consenting to marriage, one also give consent to sex. One young woman had this to say:

*The emphasis was on the bedroom issue, as it's the core reason people get married. They said that, never refuse your husband every time he wants you, If you refuse him, he will go out looking for other women (*Zione, a 24 year old young woman).

#### 4.1.4 Personal and societal identity

Among the Yao people, the celebration of traditional initiation rites serves as a powerful unifying force across generations, reinforcing both personal and communal identity. These ceremonies are highly valued, with families investing significant resources to ensure their daughters participate. Failure to attend can lead to social stigma, as it may be perceived as a sign of poverty or neglect. Tradition dictates that cultural practices such as *Mzondo, Ndakula*, and *Litiwo* are passed down through generations. As one young woman, Mana (23 years old) explained:

*The tradition here is that if your parents went through all the cultural practices like Mzondo, Ndakula and Litiwo, you must go through all the cultural practices. In my case, I went through, all the cultural practice, my mom also went through the same, so my children will also go through all the cultural practices*.

Initiation rites also play a crucial role in shaping young people's identities and elevating their social standing within the community. During these ceremonies, initiates are introduced to various rituals and riddles, which they are expected to memorize and master. Mastery of these traditions distinguishes those who have completed the process from those who have not. Participation in these rituals is considered an honor. Dunia, a grandmother over 80 years old, shared

*When I travel somewhere, for instance, when I go to Liwonde [a neighboring town] and there is an initiation ceremony, and I want to attend, they will ask me at the entrance, what did you see? “I saw this and that, this, and that, this, and that.” They will know you have gone through the process, then you will hear ululation, signifying being accepted to participate in the ceremony*.

Amina, a 24-year-old young woman also said:

“*We are encouraged as Yao people not to abandon our culture and its traditions, as such the practices of Mzondo, Ndakula and M'meto [Litiwo] are being encouraged to go forward.”*

Through these rites, individuals gain not only a sense of belonging and respect but also a deep connection to their heritage and community.

#### 4.1.5 Intergenerational use of health knowledge systems

Findings further revealed that all three generations, young women, mothers, and grandmothers, relied on both traditional and Western medical systems, selecting whichever approach was perceived as most beneficial based on the situation and the perceived cause of illness. Clear generational differences however also emerged. Younger women tended to favor Western medicine and modern health practices, while older women placed greater emphasis on traditional medicine, indigenous knowledge, and associated rituals. This divergence often created tension between generations. Older women expressed concern about the erosion of cultural identity, history, and beliefs, whereas younger women appeared less committed to preserving these traditions. One mother said:

*Today's youngsters have abandoned the old practices as they feel that when they attain puberty, they just need to be told on how to wear pads, they think that this is enough. They are refusing the old practices, yeah where they could learn this and that. If they had accepted, to go through these cultural practices, they could be disciplined, and they would know the consequences of their decisions and behavior* (a 50-year-old Mother: Esime).

Esitere a 70+ year old grandmother said:

*We are seeing many young people not wanting to follow the tradition. Only very few people are sending their children to Mzondo. Those who have been at Mzondo are well mannered, are afraid of engaging in bad behaviors as they always remember what they were told at Mzondo. But those who have not been at Mzondo are not disciplined*.

Alima a 65-year-old key informant added:

*But the children we have given birth today, they have brought bad practices, they say their children cannot go to Mzondo, because there are sins at Mzondo because of their Islamic teaching* (Alima).

Despite these differences, both knowledge systems were recognized as valuable in their own ways. In response to the issues identified during the preliminary analysis, a health improvement intervention was deemed necessary to bridge the generational gap and to integrate modern SRH education with traditional practices, addressing harmful elements while preserving beneficial cultural knowledge. Table 2 outlines the adaption phases, questions, methodological decisions, results, and observations.

### 4.2 Phase 2: decision

This step involved determining what evidence-based intervention will be selected and deciding whether it will be adapted or adopted. We conducted a community meeting and shared the preliminary findings with the traditional SRH counselors and young women and assisted them to identify issues contributing to teen pregnancies and come up with possible solutions. The first author and the community partner met the two groups separately, starting with the traditional SRH counselors. Each group was asked to do a mini evaluation of the counseling process of the traditional initiation ceremonies, the content, the aims of the rituals and practices, and the benefits to the girls. After that, the first author presented the findings highlighting the strength and areas that she felt needed improvement. At first their responses demonstrated that they were unable to see the link between the initiation rites, and the teen pregnancies or risky sexual behaviors in general, stating that the girls were fairly guided during the initiation ceremonies. But when the question was rephrased depicting the issue as a general problem and how it could be solved, the team leader was quick to suggest that they should encourage the girls to go for contraceptives and stop using the traditional contraceptive method of tying a string around the waist to prevent pregnancy. One traditional SRH counselor said: “*traditional contraceptives are no longer effective these days. Some women these days become pregnant while the strings (traditional contraceptives) are around their waists.”* Thus, the traditional SRH counselors accepted and acknowledged the existence of the problem and identified solutions to the problem, as well as challenges associated with changing that reality. Together we reflected on the relationship of the initiation ceremonies and the teenage pregnancies which was identified as the main sexual health problem in the village and discussed problems/ issues which were contributing to teenage pregnancies as well as solutions. The team agreed to leverage the traditional initiation ceremony, organize the teachings, and include the scientific evidence-based information.

Based on the literature review conducted, the team agreed to incorporate the Malawi Family planning guidelines (48), into the initiation ceremony curriculum. Together with the community partners and the traditional SRH counselors, we deliberated on the logistics of adding new content to the counseling sessions during the initiation ceremonies. We agreed to introduce the intervention to *Ndakula* the 1-week puberty rite for young girls. This seemed the most appropriate because, there was enough time for a group or individual motivation talks on family planning/ contraceptives. We also agreed to focus the talk on the benefits of short-term contraceptives, with emphasis on condoms because of their dual protection and where to find them, if interested. Participants expressed concerns about how the plan would be implemented, given their limited knowledge of modern (Western) contraceptives. We assured them of our commitment to provide comprehensive support, including information, training, and necessary materials. The Principal Investigator (the first author) a Professional Community Health Nurse and Community Health Nurse Lecturer at a national institution of higher learning, along with the HSA, who served as a community research partner, had direct access to government family planning resources. Their familiarity with the health system positioned them well to make appropriate referrals and ensure that family planning resources were available and accessible to the girls. Additionally, to further increase access to family planning services, the HSA is authorized to provide short-term contraceptives such as pills, injectables, and condoms. This further reassured participants of the consistent availability of contraceptive options within the community. Thus, it was agreed that the main role of the traditional SRH counselors during the puberty rite would be to advise the young women on how to prevent pregnancy and to refer them to the HSA if they showed interest for more counseling and provision of the chosen method.

### 4.3 Phase 3: adaptation

In this phase, we employed an innovative pretesting approach known as theater testing (31) to adapt the traditional puberty initiation ceremony (*Ndakula*). Ten young women were invited to participate as actors, girls who have just attained menarche and were being initiated, while traditional SRH counselors demonstrated the key elements of the ceremony, with a particular focus on the counseling component. The primary aim was to identify aspects of the ceremony that required adaptation. Key stakeholders, including community health nurses, two HSAs in the catchment area, and other influential village members attended as observers. Following the demonstration, all participants engaged in a focus group discussion facilitated by the first author to review the counseling content, determine necessary adaptations, and discuss strategies for implementing these changes. The group identified controversial areas and elements that could potentially encourage early sexual initiation and pregnancies. Together, they brainstormed appropriate information and specific language to ensure the content was both culturally sensitive and supportive of positive sexual and reproductive health outcomes.

### 4.4 Phase 4: production

During the production phase, we produced the first draft of the adapted intervention, which was named Partnership in Action: Health Improvement Intervention (PAHII). We incorporated feedback from focus group discussions during theater testing and with traditional SRH counselors and young women determined how to maintain the fidelity of the puberty traditional initiation ceremony. This collaborative input guided the creation of the initial formal draft. Consistent with evidence-based intervention adaptation literature (32), we retained non-harmful core elements of the traditional initiation ceremony to preserve cultural fidelity. These included: the calabash ritual, where girls break a calabash with the right foot to symbolically shield them from harm and render them invisible to those with ill intentions, including witches; instruction on marital roles, particularly how to care for future husbands; and the water ritual, which involves diving into water with a tight fist holding maize flour, ensuring it stays dry upon surfacing before throwing it into the air, signifying full cultural initiation and eligibility to attend future *Ndakula* ceremonies. Menstrual hygiene education was also preserved, emphasizing secrecy, the perceived dangers of menstrual blood, and abstaining from sexual activity during menstruation. Girls were taught how to use traditional sanitary pads and were given two sets of beads (red and white) to discreetly inform their husbands in future about menstruation by hanging them on the wall or placing them on the sleeping mat.

Other retained components included sexual health advice. This focused on abstinence to prevent unplanned pregnancies and STIs, including HIV/AIDS; domestic responsibility such as helping elders with chores; teachings on respect for community members to promote social harmony; the practice of self-elongation of the labia minora (known as “elevens”), a traditional belief with no proven health benefits, but believed to protect the vagina, likened to how every house needs a fence; and communal celebration rituals involving eating, dancing, and drinking local sweet beer at the riverbank. We worked closely with the traditional initiation counselors and the community health workers to make sure that the unharmful core elements are retained. However, we added content about female reproductive system, menstrual cycle, including ovulation and fertility awareness, family planning methods and sexual and reproductive health and rights.

### 4.5 Phase 5: topical experts

In Phase 5, the senior author, a Malawian expert in women's sexual and reproductive health, maternal and child health, adolescent health, community-based participatory research, and implementation science research was engaged as a process and content expert. Her responsibilities included guiding the adaptation process, identifying traditional practices that were culturally significant yet non-harmful, and advising on language choices to maintain cultural authenticity. This expert involvement ensured that the adaptation process was both culturally sensitive and scientifically rigorous. The second author, a Medical Sociologist with decades of experience researching the social dimension of long-term illness including HIV and AIDs, attitudes, and stigma and Dr LN, a Senior Lecturer in the School of Health and Social Care, UK who both supervised the whole research process provided on oversight of the adaptation process. Their international perspective and expertise were critical, ensuring that the adaptation was methodologically sound and met the highest standards of scientific integrity. Feedback from these experts informed revisions to the intervention curriculum, enhancing its relevance and accessibility for Yao traditional SRH counselors and adolescent girls. Additionally, the experts contributed to the development of interview questions and the selection of methods to assess the intervention's effectiveness, feasibility, fidelity, and sustainability.

The senior author was invited as a process and content expert to support the adaptation process, and in identifying culturally significant yet non-harmful traditional practices and advising on the appropriate language to preserve cultural authenticity and ensured that the adaptation process remained both culturally sensitive and scientifically rigorous. The second author also provided an outsider oversight to make sure the adaptation remained scientifically rigorous. Feedback from the experts informed revisions to the intervention curriculum, making it more relevant and accessible to Yao traditional SRH counselors and adolescent girls. Additionally, the experts contributed to the development of interview questions and the selection of methods to assess the intervention's effectiveness, feasibility, fidelity, and sustainability.

### 4.6 Phase 6: integration

The integration phase involved integrating content provided by the topical experts into the adapted draft of the intervention (31). The integration of content from the topical experts resulted in the completion of the second draft of the adapted traditional initiation ceremony curriculum. Integration of intervention content suggested by the topical experts was weighed by prioritizing the capacity of the traditional SRH counselors to implement the suggested adaptation and by maintaining fidelity to the core elements and theoretic underpinnings of the original traditional initiation ceremony curriculum. We also incorporated readability testing using the Flesch-Kincaid readability test (49), a computerized tool for assessing grade-level reading skills that is locally accessible online. This ensured that our materials were tailored to the literacy levels of traditional SRH counselors, most of whom had no formal training. Additionally, the training manual was translated into the local language and designed with a strong emphasis on visual content, using pictures to enhance understanding and accessibility.

### 4.7 Phase 7: training

**Figure 2:**
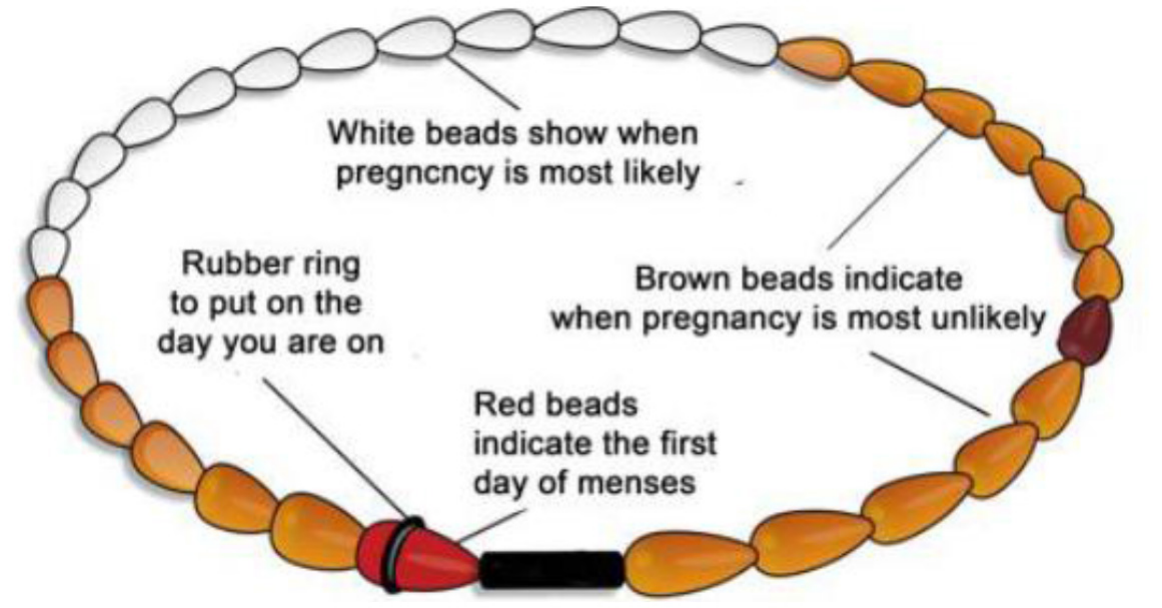
Beads that shows when a girl/woman can or cannot become pregnant (48).

In this phase we trained traditional SRH counselors, young women, and the HSA to implement the adapted sexual health intervention. This step involved addressing some of the problems and gaps in knowledge identified during the preliminary analysis through training sessions delivered to the traditional SRH counselors and the young women. To enhance the learning process, we relied on visual teaching aids which were adapted to the local needs to increase intervention uptake and success, and for easy understanding, as most of them could not read and write. Although the content was almost the same, there was a slight difference in the way it was presented and handled to the two groups, taking into consideration their ages and the societal norms. For the young women we revised the existing content to focus more on empowerment, avoiding unwanted sexual situations and choosing healthy relationships. With the aid of red, brown, and white beads (depicting the three phases of the menstrual cycle as normally used during the traditional initiation ceremonies) the trainer (the first author) taught them about menstrual cycle, ovulation, and the fertile days of a woman, starting with what happens when a girl attains puberty, and showing them the female reproductive organs to aid understanding. Figure 2 shows the Cycle beads.

#### 4.7.1 Checking the knowledge following the trainings

After the training, the participants were asked to evaluate the training session. We conducted a focus group discussion. Questions included: *What have you learned from the training*? W*hat did you like most about the training? What is it that you liked the least? What would be your recommendation?* There was positive feedback from both groups. The traditional SRH counselors specifically mentioned that, they liked the use of pictures most, saying it made the lessons interesting and easier to follow. They went further to suggest that we should initiate a similar project in the neighboring villages, so that, they benefit too. On the other hand, the young women commented that; if they had had this information then, they would not have had unplanned pregnancies, and that they would not want others to go through what they went through.

### 4.8 Phase 8: testing

This stage involved putting into action the adapted intervention (PAHII), and enable the traditional SRH counselors to test the intervention on real younger girls who just attained menarche. The first author in collaboration with the HSA and traditional SRH counselors tested the adapted curriculum, the PAHII on two girls who had just attained menarche. At this session, the local community health worker, the HSA, and the young women were designated as the observers of the testing process. After the session, the team conducted a focus group discussion, to discuss the experiences of the SRH traditional counselors, and determine what needed to be refined. The first author and the HSA met with the two groups (the traditional counselors and young women) separately, starting with the traditional SRH counselors to do a focus group discussion. The team evaluated the counseling process, the content, the aims of the rituals and practices, and the benefits to the girls, and discussed areas which went well and those that needed improvement. Both groups welcomed the intervention. One traditional SRH counselor had this to say:

“*You should have taught us this, the first time you came to this village, and by now all these girls we have taken to the initiation camps would have known about these issues*.” (Anagama, a 72 year year old traditional counselor).

The intervention was evaluated one year after of its implementation through a descriptive assessment. This included reviewing the family planning register for the catchment population, conducting informal interviews with new initiates, and observing an initiation ceremony to determine whether new knowledge had been integrated and harmful practices removed from the curriculum. The results were promising, showing positive ripple effects that benefited all women of reproductive age. In Malawi, each community health worker, known as a Health Surveillance Assistant (HSA), maintains a register of all individuals in their catchment area. In 2022, data from the Mbatamila Village Health Register, where the study and intervention were conducted, recorded 303 women of reproductive age (15–49), with 27 reported pregnancies before the intervention. By 2023, following the intervention, the number of pregnant women had decreased to 13, representing a 51.9% reduction. Similarly, the number of women using family planning methods increased from 87 in 2022 to 159 in 2023, an 82.8% rise after the intervention.

## 5 Discussion

This paper describes the systematic adaptation of a culturally accepted traditional initiation ceremony of the Yao tribe in Malawi into a SRH intervention for young women following puberty. The adaptation followed the ADAPT-ITT framework, which proved to be both clear and effective across all eight phases. In its first year of implementation, the intervention, PAHII, demonstrated strong community acceptance. It contributed to increased utilization of family planning services and a noticeable reduction in unplanned pregnancies in rural Balaka, where the intervention was implemented. This increase was attributed to the new content which had been added to the initiation ceremonies and the training which the traditional SRH counselors and young women had gone through.

The results affirm the cultural relevance and acceptability of the intervention content among both traditional SRH counselors and the young women who participated. Notably, these counselors continue to integrate the adapted intervention into their initiation ceremonies, reinforcing the sustainability and cultural integration of PAHII. These outcomes underscore the intervention's potential to reduce early pregnancies among Yao girls, who have historically faced high rates of early childbearing following initiation rites. These findings are consistent to what Chandler et al. (50) reported that in addressing the SRH issues for racial and ethnic groups, there is a need for the development of innovative programs, framed using theoretical underpinnings that are culturally and contextually tailored so that they align with lived experiences. Future SRH interventions targeting culturally rooted practices should consider culturally sensitive adaptation frameworks like ADAPT-ITT. This approach not only enhances acceptability but also improves health outcomes in traditionally underserved populations. In each phase of the adaptation process, we incorporated the principles of participatory action learning of reflect, plan, act and observe (36). Incorporating the principles of reflect, plan, act, and observe into each phase of the ADAPT-ITT model significantly enhanced the model's capacity to deliver culturally grounded, responsive, and sustainable health interventions. These four principles formed a continuous learning cycle that strengthened each phase of the adaptation process when working in a culturally underserved community. By pausing to reflect on cultural norms, community needs, past interventions, and the socioeconomic context, the participants were better equipped to ensure that the adaptation is not only evidence-informed but also culturally appropriate. This critical thinking helped avoid superficial modifications and instead fostered meaningful alignment with the target community's lived realities. The principle of planning was essential across all phases, as it ensured that the adaptation process was intentional, structured, and inclusive.

Planning helped define clear goals, roles, timelines, and communication channels; and provided a roadmap for how the traditional initiation ceremony curriculum would be reshaped while maintaining its core components. Action brought the planning and reflection to life. In the Integration and Training phases, stakeholders move from conceptual adaptation to tangible implementation. Incorporating the principle of action here ensures that the intervention materials, training programs, and delivery mechanisms are tested in real-world contexts. It also provides opportunities to empower local facilitators and traditional SRH counselors to take ownership of the intervention. Finally, the principle of observation was integral during the testing and implementation phases. Through careful monitoring and evaluation, the research team assessed how the intervention was received and the outcomes. Observation created a feedback loop that informed further reflection and refinement, ensuring the intervention is effective and relevant over time. Together, these four principles reinforced the ADAPT-ITT model's strengths by embedding a participatory, iterative, and context-sensitive process throughout each phase. They ensured that adaptation is not a one-time technical activity but an ongoing, reflective, and community-driven practice. This approach is particularly critical for sexual and reproductive health interventions that operate at the intersection of tradition, identity, and public health.

### 5.1 Lessons learned

**Lesson 1: Cultural rituals and traditions among ethnic groups serve as powerful vehicles for conveying SRHs information to girls and young women**.

Among Yao culture, traditions, and rituals often rich in symbolism, carry generational knowledge that shape social norms and individual behavior. When unharmful, these practices present valuable entry points for delivering health messages and can be strategically leveraged to enhance the cultural relevance and acceptance of sexual and reproductive health interventions. For example, the symbolic act of drawing red, black, and white lines on a girl's thigh, used to signal the presence and nearing end of her menstrual period, holds significant meaning in communicating menstrual taboos and expectations. Building on such culturally embedded practices, the PAHII developed incorporated similar rituals to educate young women about the reproductive cycle and the appropriate timing for contraceptive use. Learning in this context becomes more meaningful through the connection between knowledge, shared cultural practices, and lived experiences. The intervention revealed that many young women became pregnant not because of defiance, but due to a lack of accurate information about their reproductive health and contraceptive options. This insight aligns with findings from Munthali et al. (4) who observed that initiation rites among Yao women in Machinga District, southern Malawi, often failed to provide comprehensive and accurate SRH information, thereby contributing to early pregnancies. Our intervention incorporated scientific content on the anatomy and physiology of the reproductive system, ovulation, fertility awareness, and contraceptive options into the traditional initiation curriculum. Integrating this information was essential, as it strengthened counselors' capacity to provide accurate, evidence-based guidance that could help girls make informed decisions and prevent early pregnancies. This approach bridged traditional knowledge with scientific understanding, fostering culturally grounded yet health-promoting education. It also aligns with Wallerstein and Duran (22), Greenhalgh et al. (23) who emphasize the importance of integrating stakeholder perspectives and co-developing interventions that are both contextually relevant and scientifically sound. newline

**Lesson 2: Traditional initiation ceremonies are critical in the fight against early pregnancies, HIV and other STIs**.

This study found that open discussions about sexual health in rural Balaka were largely restricted to cultural initiation ceremonies. In societies where sexual conversations are regarded as taboo, initiation rites provide a rare, socially acceptable space for such topics to be discussed freely and openly. Additionally, the secrecy surrounding these ceremonies is deeply rooted. Individuals who have undergone the rites are often unwilling to share what they learned with those who have not yet been initiated. Instead, they encourage others to participate in the initiation themselves, suggesting the reason why nearly every girl, parent, or guardian expressed support for the puberty rite, with many parents even saving money to ensure their daughters could attend. Also, despite occasional proposals to ban such traditional practices, many communities continue to uphold initiation ceremonies because they are perceived as crucial to cultural identity and social belonging. Similar findings were reported by Talakinu (51) in a study on *Chinamwali* (traditional initiation ceremony) among the Bemba in Zambia. Talakinu noted that women who had not undergone initiation were viewed as unfit and uncultured, highlighting the ceremony's role in conveying expected societal norms and behaviors.

Given their cultural importance and influence, these ceremonies present a valuable opportunity for delivering accurate and age-appropriate SRH information. Training traditional SRH counselors to incorporate essential SRH topics, such as the reproductive cycle, gender dynamics, and contraceptive use, can enhance the relevance and impact of the teachings. This is particularly important because, although sexual abstinence before marriage is strongly encouraged on moral and religious grounds, broader public health perspectives on issues like unintended pregnancy, STIs, and HIV/AIDS are often underemphasized. Traditional SRH counselors, without sufficient training, may instead rely on outdated or inappropriate methods of instruction, such as demonstrating sexual moves, leaving young women vulnerable and ill-equipped to make informed decisions. This study demonstrated that when SRH counselors and young women were equipped with accurate SRH knowledge and skills integrated into the initiation ceremony, the results were significant. The findings support the need to leverage traditional initiation practices as culturally acceptable platforms to address early and unplanned pregnancies, HIV, and other sexually transmitted infections. Talakinu (28) also recommends the integration of transformative messages in the traditional initiation ceremonies curriculum that could empower the initiate to confront harmful cultural practices. Although training traditional initiation counselors was a key strategy to promote fidelity in implementing the adapted intervention and to prevent the reintroduction of inaccurate or harmful messages, a more rigorous evaluation is needed to assess both the long-term sustainability of the intervention and the consistency of its delivery over time. newline

**Lesson 3: The power of oral communication**.

This study highlighted the enduring power of oral communication over written forms of knowledge transmission in the context of SRH education. Oral communication, often perceived as informal is a foundational method through which knowledge, beliefs, traditions, and values are acquired and passed down across generations, typically mediated by elders who act as living repositories or libraries of communal wisdom (52). The effectiveness of oral tradition in interpreting, preserving, and transmitting the thought systems and philosophies of African peoples was clearly evident in this study. Traditional SRH counselors learned their roles through oral apprenticeship from parents and elder relatives, rather than formal schooling. During initiation ceremonies, for example, the counselors operated without a written curriculum, yet demonstrated a structured, consistent, and culturally nuanced delivery. They knew what to teach, when to teach it, and who was responsible for delivering specific messages. This supports Mokgobi (53)'s findings on how traditional healers acquire competence through oral instruction and practice. The role of the traditional SRH counselors therefore goes far beyond providing advice on menstrual hygiene or sexuality. These individuals are custodians of indigenous knowledge systems. Their respected status in the community, even among village chiefs, underscores their cultural authority and social significance. This study affirms the value of indigenous wisdom and demonstrates how it can be integrated into sustainable SRH strategies. By merging indigenous systems of knowledge with conventional Western approaches to sex education, interventions can become more culturally responsive and impactful. Such an approach promotes decoloniality and African feminism and contributes to the transformative educational paradigms currently emerging from the African context (28). newline

**Lesson 4: Local problems require locally grounded solutions**.

Another key lesson emerging from this study is that health challenges, particularly those as sensitive and culturally embedded as SRH cannot be effectively addressed through top-down, one-size-fits-all approaches. Instead, sustainable change is most likely when interventions are co-designed with the communities they intend to serve. This study demonstrated that by engaging community members, particularly traditional leaders, elders, and SRH counselors as equal partners in changing some risky traditional practices and messages embedded in traditional initiation rites, culturally appropriate and socially acceptable strategies can be developed. Community engagement fosters ownership, relevance, and trust, all of which are critical to the success of health interventions consistent with previous research (18, 22, 54). By recognizing and leveraging existing cultural frameworks, such as initiation ceremonies, communities become active agents in shaping solutions that align with their values while addressing public health goals. This participatory approach not only enhances the effectiveness of interventions but also contributes to community empowerment and resilience (18, 22, 54).

The power dynamics within the participatory research process could not be overlooked in this study. The first author's positionality as an educated health care professional and University Lecturer working among local young women, mothers, and grandmothers inevitably introduced elements of power and privilege. Acknowledging this, she approached the research with a commitment to co-creating knowledge and recognized the fluid and evolving nature of her relationship with participants. To mitigate these dynamics, an Ethnographic Participatory Action Research approach was adopted, and the researcher lived in the study village for nearly two years. This prolonged engagement fostered trust, rapport, and familiarity, allowing her to move from being perceived as an outsider to becoming an accepted member of the community. Throughout the process, LYC maintained a reflexive logbook to document her thoughts, emotions, and interactions, critically examining how her background, assumptions, and social position influenced data collection and interpretation. Power relations were not static but continuously negotiated. During participant observation, the researcher assumed the role of a learner, gaining insight from rural women, particularly traditional SRH counselors about cultural practices and social meanings surrounding sexual and reproductive health. Her role later shifted to that of a facilitator during the co-development and implementation of the health improvement intervention. This transition reflected a deliberate effort to redistribute power, valuing participants as co-researchers and knowledge holders. Such reflexivity and role fluidity helped minimize hierarchical boundaries and strengthened the participatory integrity and methodological rigor of the study.

### 5.2 Study limitations

This study has some limitations that should be considered when interpreting the findings. First, the intervention was conducted in a single community within a specific cultural context among the Yao people, and at a relatively small scale. As such, the findings are not generalizable to all rural Yao women or to other ethnic groups in Malawi or sub-Saharan Africa. Future studies should replicate this intervention in diverse settings and among various cultural groups to explore contextual adaptations and improve generalizability. Second, the study relied exclusively on qualitative methods which provided rich contextual insights into cultural dynamics. The absence of a quantitative component limits the ability to measure the extent of changes in key outcomes such as contraceptive knowledge, uptake, and incidence of early pregnancy before and after the intervention. Incorporating mixed method approaches in future studies would allow for more robust evaluation of the intervention's impact. Furthermore, the short time frame of the study limits the ability to assess the long-term outcomes and sustainability of behavior change. Building on these findings, future studies should employ a larger, longitudinal mixed methods study design to evaluate how the intervention influences outcomes over time, whether cultural integration can be maintained, and how community norms continue to evolve. Integrating quantitative measures such as pre- and post-intervention surveys with in-depth interviews and focus groups to triangulate findings and assess impact more rigorously, and determine how to scale and adapt the intervention across multiple geographic and cultural settings to evaluate its feasibility, cultural adaptability, and cost effectiveness, as well as to determine the sustainability and scalability of integrating SRH into traditional rites of passage potential for broader implementation. Third, the final testing (phase 8), an essential component of the adaptation process, was conducted with only two adolescent girls who had recently attained menarche. Although their feedback was useful, the small sample size limited the depth and diversity of perspectives. A larger sample size would improve understanding of intervention fidelity, content relevance, and participant engagement. Another limitation of this study is that the evaluation conducted one year after the intervention was descriptive in nature. Informal interviews were held with new initiates, and data from the village health register were used to assess changes in the number of women becoming pregnant and those using family planning methods. Although positive outcomes were observed, it is difficult to attribute these improvements solely to the intervention. A more rigorous evaluation is recommended, incorporating a mixed-methods design with both quantitative and qualitative components. This could include pre- and post-intervention surveys using standardized instruments, longitudinal follow-up to assess sustained impact, and comparison or control groups to strengthen causal inference. Additionally, statistical analyses such as regression modeling or difference-in-differences approaches could help isolate the intervention's effects while accounting for confounding factors.

Additionally, men were not included in this study, which may influence the broader uptake and sustainability of the intervention. Given that men often hold significant influence over SRH norms and decision-making within families and communities, their exclusion represents an important limitation. Future research should therefore examine male initiation ceremonies already existing in the study setting, and strategies for male engagement, focusing on the roles of boys, fathers, and male elders in shaping, reinforcing, or challenging prevailing norms around adolescent SRH. Involving men as allies and advocates could foster shared responsibility and contribute to creating supportive environments that protect young girls from early pregnancies and promote gender-equitable SRH outcomes.

It is also essential that future research should formally include adolescent girls' feedback as a core component of the evaluation process to center their voices and experiences in both formative and summative assessments. There is also a need to explore male engagement, particularly the role of boys, fathers, and male elders in reinforcing or challenging norms around adolescent SRH, to ensure that males also protect the young girls from early pregnancies. By addressing these limitations and exploring new avenues of research, scholars and practitioners can strengthen the evidence base for culturally grounded SRH interventions and advance more inclusive, locally resonant strategies to improve the health and agency of young women.

### 5.3 Implications

This study has important implications for practice, policy, research, and education. For practice, health care providers in Malawi can use the proposed model to mobilize and train traditional initiation counselors across the country. By equipping counselors with accurate, culturally sensitive SRH information, they can be empowered to educate girls during initiation ceremonies and transform these traditional spaces into effective SRH learning platforms. Traditional community health nurses and Health Surveillance Assistants can also collaborate with counselors to strengthen community-based efforts aimed at preventing teenage pregnancies, HIV, and other STIs. For policy, while the Malawi government recognizes the importance of community participation in improving health outcomes (55), there are currently no formal policies that integrate local knowledge holders into the health system. This study provides a practical example of how traditional structures can be meaningfully engaged in health promotion. The findings can inform the development of policies that institutionalize partnerships between the health sector and community actors, ensuring that local voices and cultural practices are incorporated into national SRH strategies. For research, the study highlights the need for further investigation into the feasibility and sustainability of collaborations between government health programs and traditional counselors. Future studies could evaluate the long-term impact of such partnerships on SRH outcomes, explore mechanisms for maintaining fidelity to adapted interventions, and identify strategies for scaling up community-led SRH initiatives across diverse cultural settings. For education, training institutions such as Schools of Nursing and Medicine can integrate this model into their curricula to prepare future health care providers for culturally responsive and community-engaged practice. Embedding this approach in professional education would help students develop the skills needed to collaborate effectively with traditional counselors, community leaders, and other local stakeholders. Such training would promote mutual respect, enhance cultural competence, and strengthen partnerships between the formal health system and community structures, ultimately improving the delivery and acceptance of sexual and reproductive health services.

## 6 Conclusion

This study provides important information on the process of adapting a sexual health program for early and middle adolescents and their sexual advisors and addresses an important scientific gap by developing culturally targeted, gender-specific, developmentally appropriate intervention content for a population, for whom effective sexual health interventions are lacking. Further, this study shows that community engagement can serve as a useful tool to intervention adaptation. Therefore, this study reinforces the broader principle that partnership with communities is not optional, it is essential. With genuine collaboration, even deeply rooted social and health issues like adolescent pregnancy, HIV, and limited access to contraception can be addressed in ways that are both culturally respectful and transformative. This study contributes critical insights into the process of adapting a sexual health intervention for young women and their traditional SRH counselors within a culturally rich setting. It addresses a significant gap in the literature by developing and piloting a culturally grounded, gender-specific, and developmentally appropriate intervention for a population that has historically been underserved in SRH programming. Importantly, the findings underscore the value of community engagement as a core strategy in intervention design and adaptation. By actively involving community stakeholders particularly traditional SRH counselors, elders, and young women, this study demonstrates that co-created solutions are not only feasible but also more likely to be accepted, sustained, and effective. It reinforces the broader principle that partnership with communities is not optional, but essential. With genuine collaboration, even deeply rooted cultural and public health challenges, such as adolescent pregnancy, HIV/STI transmission, and limited access to contraception, can be addressed in ways that are both culturally respectful and transformative. Furthermore, the study highlights the urgency of supporting traditional SRH counselors in preserving and documenting their indigenous knowledge. Preserving this knowledge is not only a matter of cultural heritage but also a strategy for ensuring sustainable, contextually relevant health education. In sum, this study demonstrates that the integration of traditional practices with evidence based SRH content, through respectful, participatory processes, holds promise for improving health outcomes among young people in culturally diverse settings. Future efforts should continue to bridge indigenous and modern knowledge systems to develop inclusive, empowering, and culturally resonant health interventions.

## Data Availability

The raw data supporting the conclusions of this article will be made available by the authors, without undue reservation.
